# Polo-like kinases as potential targets and PLK2 as a novel biomarker for the prognosis of human glioblastoma

**DOI:** 10.18632/aging.203940

**Published:** 2022-03-07

**Authors:** Yiming Ding, Hanjie Liu, Chuanbao Zhang, Zhaoshi Bao, Shuqing Yu

**Affiliations:** 1 Department of Neurosurgery, Beijing Tiantan Hospital, Capital Medical University, Beijing, China; 2 Department of Neurosurgery, Beijing Neurosurgical Institute, Capital Medical University, Beijing, China

**Keywords:** polo-like kinases, glioblastoma, prognosis, biomarker, ON1231320

## Abstract

The most prevalent malignant central nervous system (CNS) cancer is glioblastoma multiforme (GBM). PLKs (polo-like kinases) are a kind of serine-threonine kinase that modulate DNA replication, mitosis, and stress responses. PLKs in GBM need to be better studied and examined in terms of their expression, function, along with prognostic significance. Using an existing publicly available data set, we evaluated the expression level and prognostic relevance of PLKs in GBM patients at the molecular level. The biological processes along with cascades of the screened gene were predicted using the functional enrichment of Gene Set Enrichment Analysis, Gene Ontology, and Kyoto Encyclopedia of Genes and Genomes pathways. The data illustrated that PLK1/3/4 contents were greater in GBM tissues than in non-tumorous tissues, but PLK2/5 expression levels were lower. PLK2 expression was also linked to patient outcome in GBM. Our findings imply that PLKs might be useful molecular indicators as well as prospective treatment targets for GBM. A PLK2 inhibitor has been studied for the first time in a glioma cell in this work. In glioma cells, ON1231320 has anticancer effects. Finally, a summary of PLK inhibitors is presented, along with projections for future progress.

## INTRODUCTION

GBM is the most frequent malignant CNS cancer in adults, which accounts for 55% of all gliomas [1]. Surgical resection, radiation and chemotherapy constitutes the current standard of treatment. However, the median time of survival is about 18 months, the survival rate is still poor [2]. Thus, treatment of GBM remains challenging. Based on high-throughput genetic, genomic, and epigenetic data, several key molecules have been identified that contribute to GBM carcinogenesis and the development of targeted therapies for individual subtypes. However, targeted therapies for specific mutations or subtypes have mostly failed due to the complexity of molecular heterogeneity within tumors [3].

Polo-like kinases (PLKs) were first found to play an indispensable role in the mitosis of *Drosophila melanogaster* [4]. Five members of PLK family have been discovered, including PLK1, PLK2, PLK3, PLK4, and PLK5 [5]. PLKs are important regulatory factors of the cell cycle in non-tumorous and cancer cells [6]. Recent advances in PLK1 research have dramatically improved our comprehension of its modulation, targets along with function. PLK1 plays an indispensable role in the regulation of the cell cycle, including activation of the APC/C (anaphase-promoting complex/ cyclosome), entry into mitosis, assembly of the bipolar spindle, sister chromatid splitting, and centrosome maturation [7]. Despite remarkable advances in the research of PLK1, the functional roles of other members of the PLK family, particularly PLK2 along with PLK3, are still unknown. PLK2 along with PLK3 have been established as key mediators of DNA damage or oxidative stress in cancer cells. A growing body of research evidence suggests that PLKs and the tumor repressor p53 interact in cancer cells [7, 8]. PLKs are aberrantly expressed in tumor cells, thereby promoting abnormal proliferation of cancer cells [9]. PLKs show aberrant expression in multiple cancers and are linked to poor prognosis. These findings have the potential to provide proof for anti-cancer medication development targets. Drug manufacturers have also invested in the research of PLKs in order to identify novel cancer medicines [6]. PLK1 inhibitors have now been tested clinically and found to be beneficial in the treatment of individuals with cancer [7].

Herein, we comprehensively purposed to evaluate the expression, function along with prognostic value of PLKs in GBM. Based on existing gene expression or copy number variants published online, we conducted a detailed analysis of PLK expression, as well as mutations in individuals with GBM. Furthermore, we performed validation using The Cancer Genome Atlas (TCGA) along with the Chinese Glioma Genome Atlas (CGGA) data resources to determine the expression, potential function, and prognostic value of PLKs in GBM.

## RESULTS

### PLK expression levels in patients with GBM

**Figure 1:**
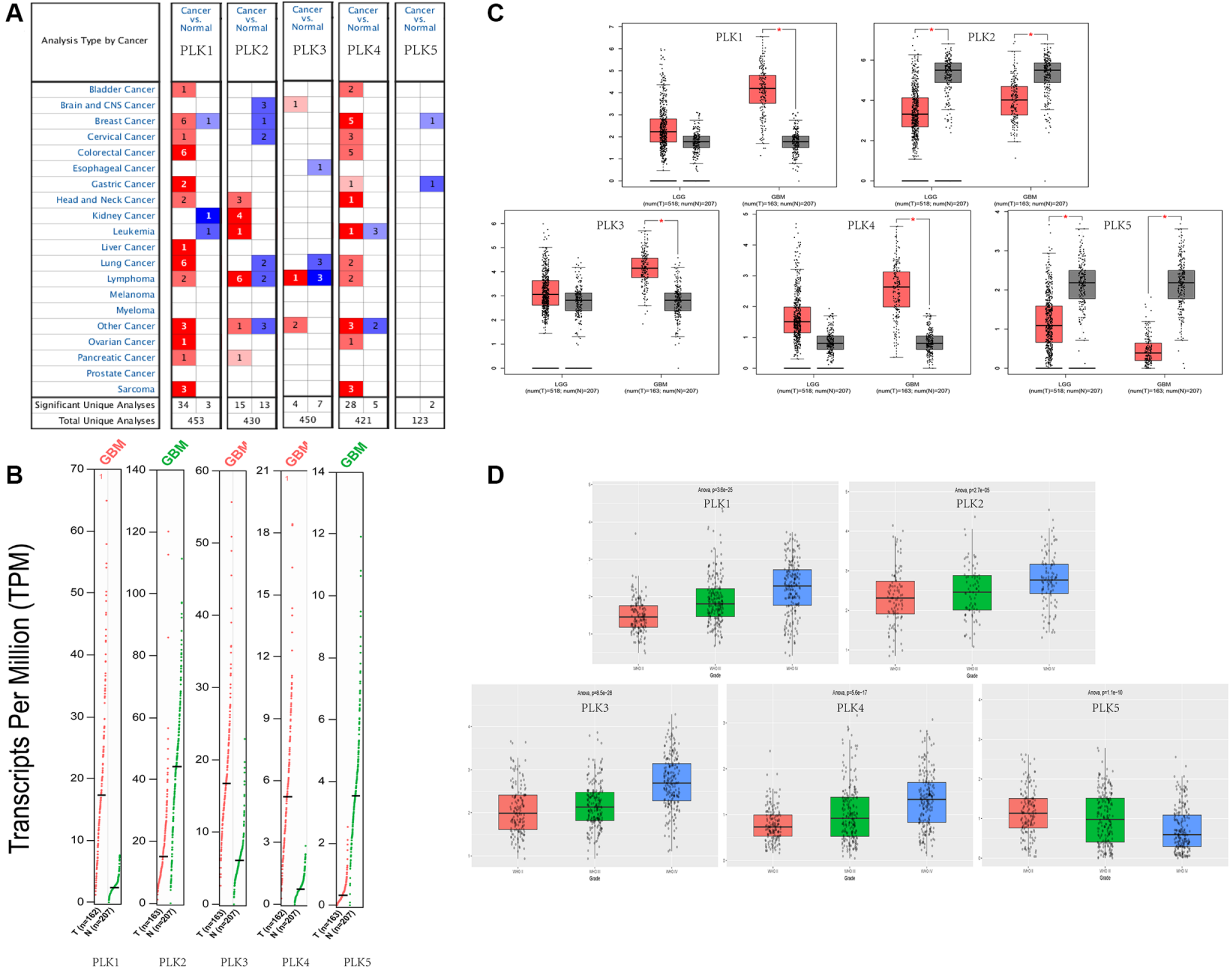
**Expression levels of PLKs in TCGA and CGGA databases.** (**A**) Expression levels of PLKs in patients with GBM. (**B**) Gene Expression Profile shows the expression of PLKs between GBM (red) and normal tissue (green) via GEPIA2. (**C**) Box plot shows the expression of PLKs between brain tumor (red) and normal tissue (grey) via GEPIA2. The “^*^” indicates that the *p*-value is statistically significant. (**D**) Box plot shows the expression of PLKs in different WHO classes via CGGA database.

We compared the expression levels of PLKs in cancer with those in non-tumorous samples via the ONCOMINE data resources (Figure 1A). The transcript expression levels of PLK1/3/4 were remarkably elevated in individuals with GBM, and that of PLK2 was remarkably downregulated in patients with GBM. PLK5 expression was not remarkably different between GBM and non-tumorous tissues. All PLK mRNA expression levels were not remarkable in low-grade glioma (LGG) tissues. The fold change and *p*-values of the PLKs are shown in Table 1. Through the Gene Expression Profiling Interactive Analysis (GEPIA2) cohort, we compared the transcript expression levels of PLKs between GBM and non-tumorous tissues (Figure 1B). The data illustrated that the contents of PLK1/3/4 were higher in GBM tissues than in non-tumorous tissues, whilst the contents of PLK2 and PLK5 were lower in GBM (Figure 1C).

**Table 1:** PLK expression levels between different types of gliomas and brain tissues (ONCOMINE database).

	**Types of glioma vs. brain**	**Fold change**	***P* value**	***t*-test**	**Ref**
PLK1	Glioblastoma	1.276	2.21E-05	6.828	Murat
	Astrocytoma	−1.073	0.674	−0.472	Rickman
PLK2	Glioblastoma	−1.7	9.03E-06	−6.079	Bredel
	Astrocytoma	2.186	0.035	2.308	Shai
PLK3	Glioblastoma	4.056	1.42E-05	4.706	Pomeroy
	Astrocytoma	−1.224	0.89	−1.353	Rickman
PLK4	Glioblastoma	1.47	4.92E-04	5.809	Murat
	Astrocytoma	−1.556	0.979	−2.257	Rickman
PLK5	Glioblastoma	1.736	0.077	1.922	Lee
	astrocytoma	1.255	0.05	1.723	Sun

As shown in the CGGA data resource, the contents of PLK1/2/3/4 rose as the World Health Organization (WHO) grades of glioma increased. However, the expression levels of PLK5 decreased as the WHO grades of glioma increased. (Figure 1D).

**Figure 2:**
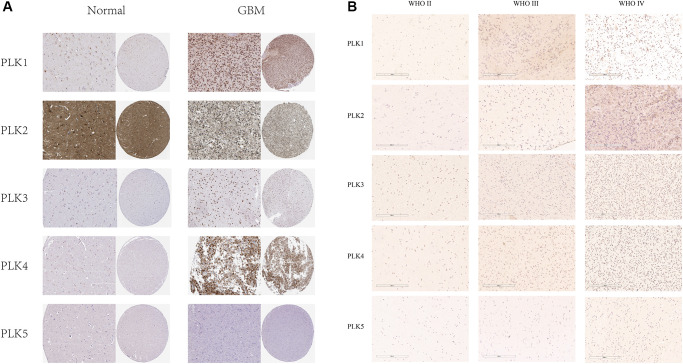
(**A**) IHC illustration of PLK expression levels in GBM (HPA). (**B**) Immunohistochemical staining of PLKs in gliomas.

Besides, immuno-histochemistry (IHC) staining abstracted from the Human Protein Atlas (HPA) data resource exhibited the contents of PLKs as shown in Figure 2A. PLK1/3/4 were over-expressed in GBM in contrast with non-tumorous tissues. On the contrary, PLK2 was more highly expressed in non-tumorous tissues than in GBM. PLK5 was expressed in neither GBM nor non-tumorous tissues (Figure 2A). Besides, immuno-histochemistry illustrated that the staining index score of PLK1-4 in AIIs was frequently less than two. In sGBMIVs, the staining index score was frequently more than four (Figure 2B). The data illustrated that the content of PLK1-4 rose in tandem with rising tumor grades, confirming our prediction.

**Figure 3:**
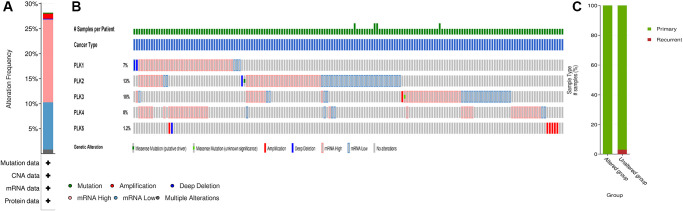
**PLK mutation analysis in GBM (cBioPortal).** (**A**) Frequency of alteration in each query in the detailed cancer types. (**B**) Overview of genetic alteration per sample in each query gene. (**C**) Explore clinical sample type comparisons among groups of samples as defined by the query.

We analyzed the PLK alterations using the cBioPortal online tool for GBM. PLK alterations included the following forms: deep deletion, amplification, mRNA high, and mRNA low. Two or more PLK alterations were seen in 29% of the samples (136 samples) (Figure 3A and 3B). In addition, using the same tool, we found that PLK alterations typically occur in the primary GBM (Figure 3C).

### Relationship of PLK contents with prognosis in GBM

**Figure 4:**
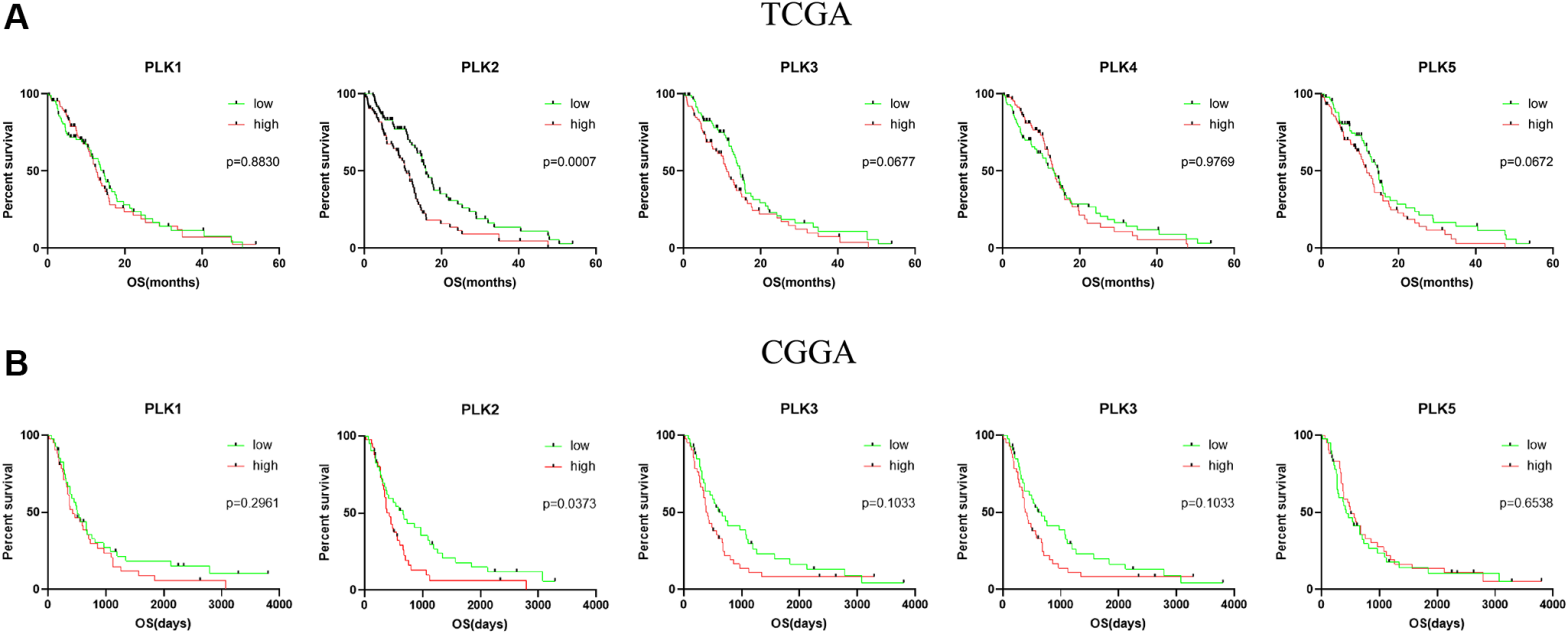
**Kaplan-Meier analysis of the prognostic value of PLKs.** (**A**) The TCGA databases. (**B**) The CGGA databases.

We further assessed the pivotal efficiency of PLKs in the survival of individuals with GBM. Kaplan–Meier Plotter tools were adopted to assess the relationship of PLK transcript contents with the survival of patients with GBM using GraphPad Prism (Figure 4A). The Kaplan-Meier curve along with the log-rank test analyses revealed that the decreased PLK2 expression level was remarkably linked to overall survival (OS) (*p* < 0.05). The individuals with GBM with high mRNA contents of the PLK2 were predicted to have poor OS. While PLK1/3/4/5 showed no prognostic significance (*p* > 0.05). This result was also validated in the CGGA data resource (Figure 4B). The high contents of PLK2 were remarkably linked to poor prognosis (*p* < 0.05). Specifically, we retrieved clinical information from the TCGA data resource, and K-M analysis showed that high PLK2 expression likewise predicted poor patient prognosis (*p* < 0.05).

We evaluated the associations between age, gender, isocitrate dehydrogenase 1 (IDH1) status, PLK expression levels, and patients’ OS in univariate along with multivariate Cox regression analyses. The univariate data revealed that age, IDH1 status, and PLK 2/3 expression were remarkably linked to patients’ OS. Multivariate analysis revealed that age and PLK2 expression were remarkably linked to patients’ OS (Table 2).

**Table 2:** Univariate and multivariate Cox proportional hazards regression analyses of prognostic factors for GBM.

	**Univariate analysis**			**Multivariate analysis**		
	**HR**	**95% CI**	***p***	**HR**	**95% CI**	***p***
**Age**						
<64	4.036	1.019–1.054	5.43E-05^*^	3.183	1.0131–1.056	0.00146^*^
≥65						
**Gender**						
Female	−0.773	0.5828–1.264	0.439	−0.747	0.5612–1.296	0.45509
Male						
**IDH1_status**						
Mutant	−2.898	0.1035–0.6455	0.00376^*^	−0.408	0.2755–2.329	0.68352
Wild type						
**PLK1**						
High	0.023	0.6902–1.461	0.982	−0.518	0.5498–1.416	0.60434
Low						
**PLK2**						
High	3.236	1.285–2.777	0.00121^*^	3.033	1.2583–2.913	0.00242^*^
Low						
**PLK3**						
High	2.033	1.014–2.164	0.0421^*^	1.564	0.919–2.123	0.11775
Low						
**PLK4**						
High	−0.202	0.6583–1.405	0.84	−0.124	0.6111–1.543	0.90136
Low						
**PLK5**						
High	1.918	0.9918–2.133	0.0551	1.429	0.8961–2.016	0.15288
Low						

### Relationship between PLK mRNA levels and GBM

**Figure 5:**
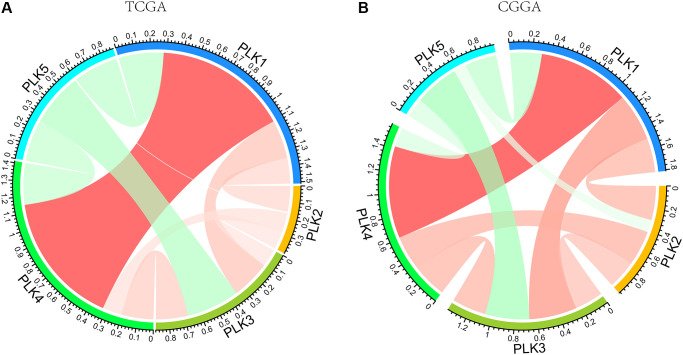
PLK correlations with each other using TCGA (**A**) and CGGA (**B**) databases.

We also computed the correlations among PLKs by analyzing their transcript expression levels via the TCGA and CGGA data resources for GBM, and Pearson’s correction was conducted. The data exhibited remarkable correlations in several PLKs. PLK5 was negatively correlated with PLK1, PLK2, PLK3, and PLK4. A positive relationship was also evident between PLK1 and PLK4 (Figure 5). As shown in Table 3, the analysis of mutual exclusivity between PLKs found that the relationship between PLK1 and PLK4 is characterized by co-occurrence (Table 3).

**Table 3:** Analysis of mutual exclusivity between PLKs (cBioPortal).

**A**	**B**	**Log2 Odds Ratio**	***p*-Value**	***q*-Value**	**Tendency**
PLK1	PLK4	>3	<0.001	0.003	Co-occurrence
PLK1	PLK3	<−3	0.114	0.568	Mutual exclusivity
PLK2	PLK3	0.7	0.324	0.9	Co-occurrence
PLK1	PLK2	0.497	0.433	0.9	Co-occurrence
PLK3	PLK4	−0.837	0.501	0.9	Mutual exclusivity
PLK2	PLK4	0	0.634	0.9	Mutual exclusivity
PLK2	PLK5	<−3	0.818	0.9	Mutual exclusivity
PLK3	PLK5	<−3	0.855	0.9	Mutual exclusivity
PLK1	PLK5	<−3	0.882	0.9	Mutual exclusivity
PLK4	PLK5	<−3	0.9	0.9	Mutual exclusivity

The analysis tested 10 pairs between the 5 tracks in the OncoPrint. Odds Ratio: Quantifies how strongly the presence or absence of alterations in A are associated with the presence or absence of alterations in B in the selected samples. *p*-Value: Derived from one-sided Fisher Exact Test. *q*-Value: Derived from Benjamini-Hochberg FDR correction procedure. Tendency: Log2 ratio >0: Tendency towards co-occurrence; Log2 ratio ≤0: Tendency towards mutual exclusivity; *q*-Value <0.05: Significant association.

### Predicted roles and cascades of the changes in PLKs factors and their frequently altered adjacent genes individuals with GBM

**Figure 6:**
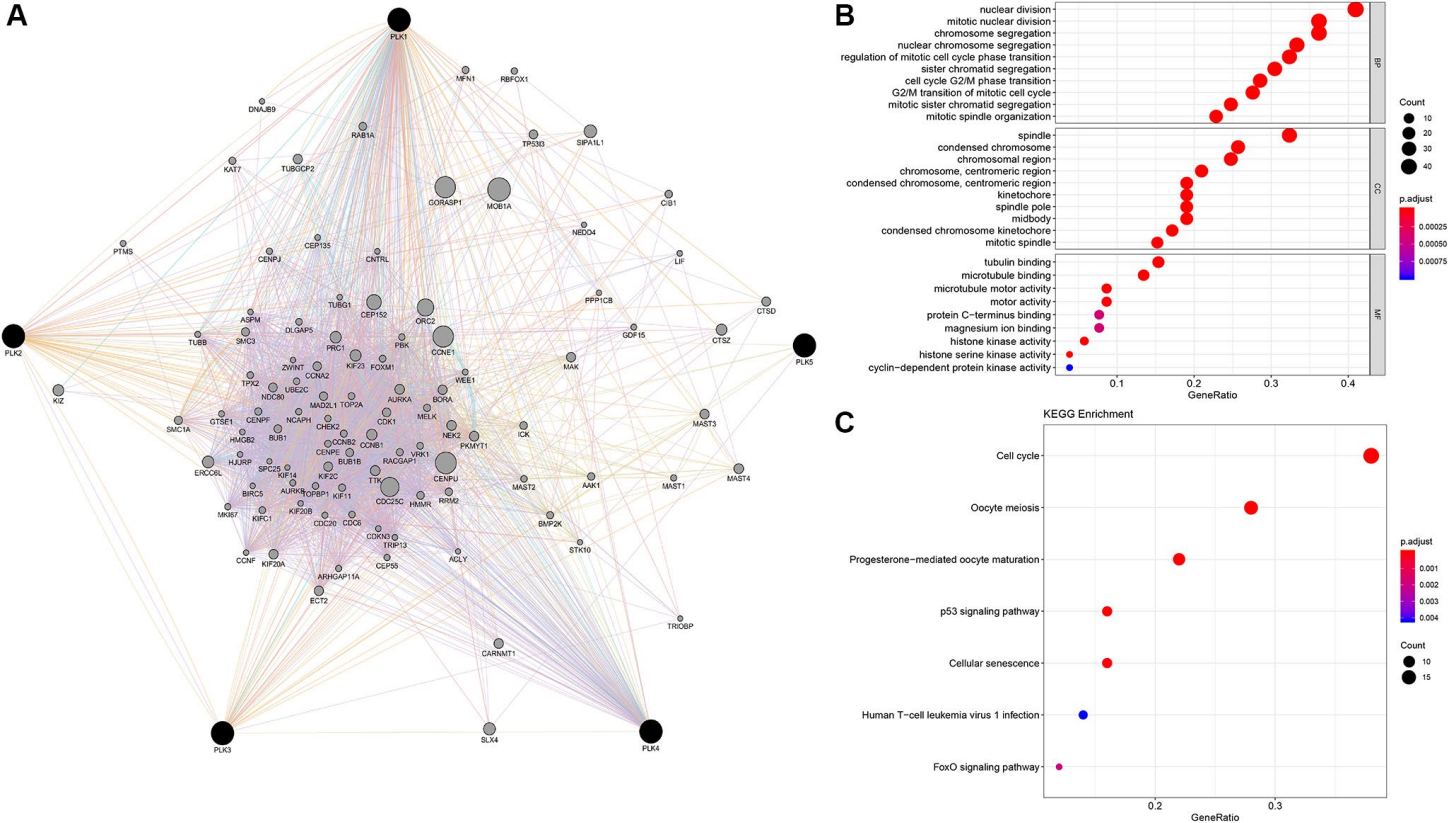
**PLK network and the functional enrichment of GO terms and KEGG pathways.** (**A**) The network of PLKs. (**B**) The functional enrichment of GO. (**C**) The functional enrichment of KEGG pathways.

Using GeneMANIA in Cytoscape, we evaluated 100 genes linked to PLKs and built a network. We explored functional enrichment of GO (Gene Ontology) terms along with KEGG (Kyoto Encyclopedia of Genes and Genomes) pathways in the network to elucidate the biological processes (BPs) and cascades of the genes. The top ten BP, CC, and MF items were chosen (Figure 6).

Nuclear division, segregation of mitotic nuclear division chromosome, modulation of mitotic cell cycle phase transition, segregation of nuclear chromosome, organization of mitotic spindle, segregation of chromatid, cell transition of cycle G2/M phase, along with segregation of mitotic sister chromatid are all BPs. The condensed chromosome in the spindle, the spindle pole midbody, the chromosomal region, the condensed chromosome centromeric region, the kinetochore, the condensed chromosome kinetochore, the chromosome centromeric region, and the mitotic spindle are all parts of the condensed chromosome centromeric region. Tubulin docking, microtubule docking, motor activity, protein C-terminus docking, magnesium ion docking, histone kinase activity, histone serine kinase activity, and cyclin-dependent protein kinase activity constituted the MFs. The cell cycle, p53 signaling cascade, oocyte meiosis, progesterone-triggered oocyte maturation, cellular senescence, human T-cell leukemia virus 1 infection, and FoxO signaling cascade were all highly enriched in the PLK network (Figure 6).

**Figure 7:**
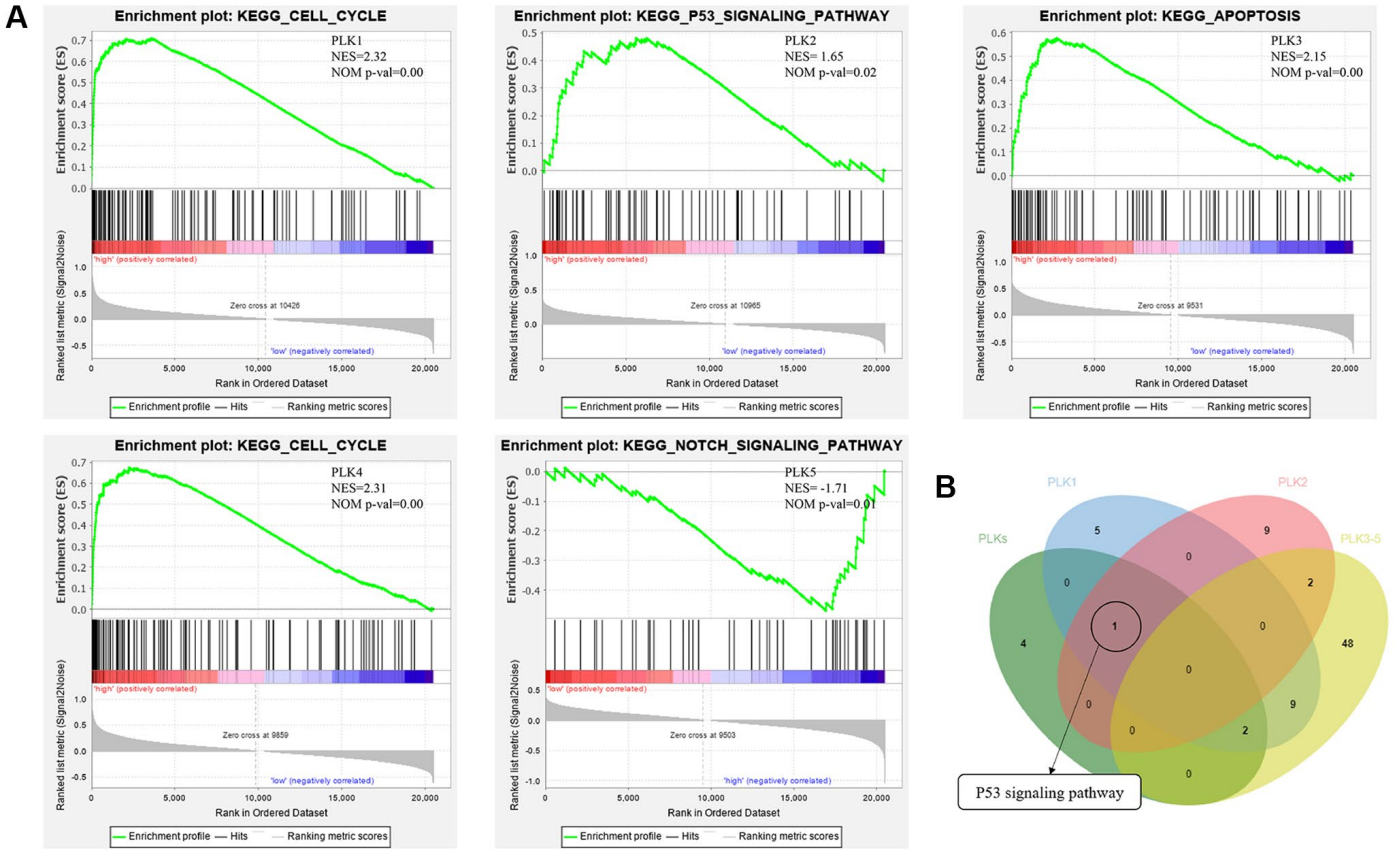
(**A**) Functional analysis of PLKs in TCGA cohort using GSEA. (**B**) Overlapping KEGG pathways between PLKs’ network and GSEA’s results.

In addition, GSEA (Gene Set Enrichment Analysis) illustrated that the high expression of PLK1 was linked to the cell cycle, the low expression of PLK2 was linked to the NOTCH signaling cascade, the high expression of PLK3 was linked to apoptosis, the high expression of PLK4 was linked with the cell cycle, and the low expression of PLK5 was linked to the NOTCH signaling cascade (Figure 7A). Finally, p53 signaling cascade was found to play a key role by overlapping KEGG cascades between PLKs’ network and GSEA’s results (Figure 7B).

### ON1231320 suppresses glioma cell proliferation and induces cleaved poly (ADP-ribose) polymerase (PARP) expression

**Figure 8:**
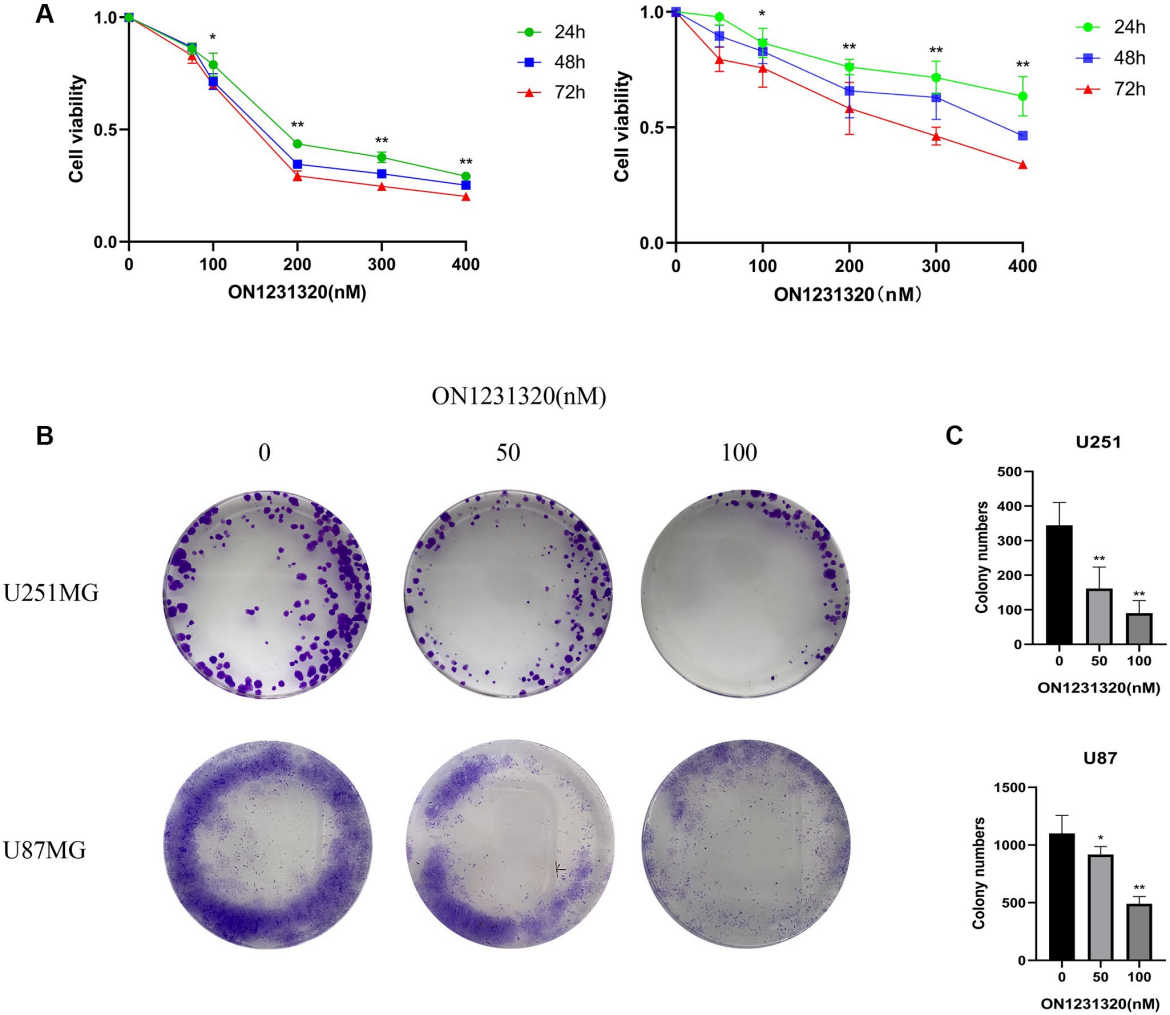
(**A**) The U251MG cells were treated under different concentrations of ON1231320 and cultured for 24, 48 and 72 h, respectively. (**B**) Colony formation assay showing the sensitizing effects on GBM cells after apcin treatment. (**C**) Quantitative results of Colony formation assay (^*^p < 0.05 vs. the control group, ^**^p < 0.01 vs. the control group).

We found for the first time that ON1231320 dampened the growth of human glioma cells. The Cell Counting Kit-8 (CCK-8) assay was done after inoculating U251MG and U87MG cells with the specified levels of ON1231320 for 24, 48 and 72 h, respectively. The data illustrated that inoculation with 200 nm ON1231320 caused remarkable glioma cell growth dampening at 24, 48 h and 72 h (Figure 8A). The colony generation of U251MG and U87MG cells after 100 nM ON1231320 inoculation was remarkably lower in contrast with the controls, and the colony generation of glioma cells diminished with increasing ON1231320 levels (Figure 8B and 8C). The data illustrated that ON1231320 remarkably dampened the growth of glioma cells in a time- along with dose-dependent approach.

**Figure 9:**
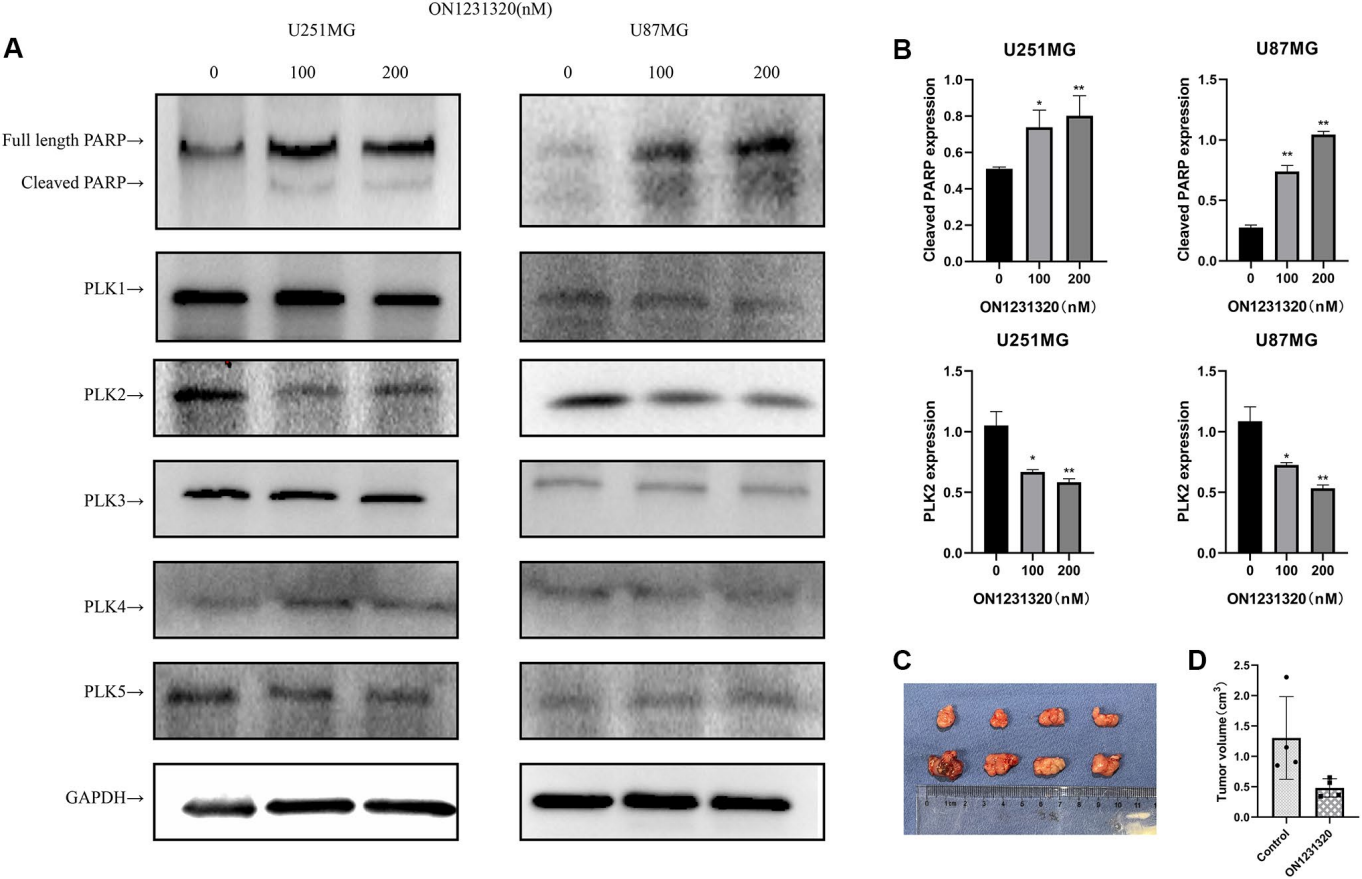
(**A**) The expression of cleaved PARP and PLKs in ON1231320 treated cells was detected using western blotting. (**B**) Quantitative results of immunoblots. (**C**) ON1231320 inhibits tumor growth *in vivo*. (**D**) Quantitative results of tumor volumes. ^*^P < 0.05 and ^**^P < 0.001 vs. the control groups.

We carried out western blot assays to further demonstrate the manner in which ON1231320 enhances apoptosis in glioma cells. The data illustrated that the expression of cleaved PARP was elevated and the content of PLK2 was decreased in U251 and U87MG cells after ON1231320 treatment (Figure 9A and 9B). While the expression of PLK1/3/4/5 was not changed. Meanwhile ON1231320 promoted cleaved PARP expression and dampened the expression of PLK2 in a dose-dependent approach. These data suggest that ON1231320 shows its antitumor properties in glioma cells.

### ON1231320 inhibits glioma cell proliferation *in vivo*

U87MG cells were subcutaneously inoculated into BALB/c Nude mice to test the effectiveness of ON1231320 *in vivo* utilizing a tumor xenograft model. ON1231320 was given daily (q.d.) through intraperitoneal inoculation at a dosage of 50 mg/kg body weight after the tumors had formed in the animals for two weeks. In contrast with the controls, ON1231320 inoculation resulted in a remarkable reduction in tumor growth (Figure 9C and 9D). ON1231320's high *in vivo* anticancer effectiveness in mice bodes well for its development for therapeutic usage.

## DISCUSSION

PLKs have been reported in common cranial brain tumors. This is the first study to explore the PLK expression along with prognosis in GBM, as well as the signaling cascades they may be involved in. It provides a diagnostic basis by contributing to existing studies on glioblastoma-targeted therapies. In addition, this paper systematically evaluates the mechanism of the PLK family in GBM and provides an important foundation for individualized GBM treatment in the future.

PLK1 is currently one of the most intensively studied molecular markers in all kinds of tumors. PLK1 is overexpressed in various cancer, including breast cancer, non-small cell lung cancer, colorectal cancer, prostate cancer, pancreatic cancer, melanoma, ovarian cancer, non-Hodgkin’s lymphomas, and acute myeloid leukemia (AML) [10]. PLK1 has also been increasingly reported in GBM. Lee et al. found that PLK1 mRNA was more highly expressed than non-tumorous human astrocytes (HAs) and identified PLK1 as a critical factor for the survival of brain cancer cells and brain tumor initiating cells (BTICs) [11]. PLK1 dampening blocked tumorsphere formation in all BTIC lines examined [11]. The present study confirmed that PLK1 is positively correlated with the malignant progression of gliomas. Likewise, Zhao et al. identified PLK1 as a key factor in the conversion of LGGs to secondary glioblastoma (sGBM) and observed that patients from the high-expression PLK1 group were more sensitive to common chemotherapies during clinical treatment. The findings of Pezuk et al. suggested that PLK1 may be a promising biomarker for the treatment of GBMs [12]. The latest research indicates that TMZ treatment activated PLK1-related signaling cascades and that PLK1 may be involved in TMZ tolerance [12]. Current small molecule PLK1 inhibitors include Rigosertib, Volasertib, GSK 461364, GW843682, PLHS-Pmab, PPG, and Poloxin [10]. In addition, PLK1 may be a tumor recognition antigen for T lymphocytes, which has important implications in tumor immunotherapy [13].

The expression of PLK2 was found to decrease with CpG methylation [14]. PLK2 methylation in ovarian cancer observed to correlate with patients treated with chemotherapy [15]. In the present study, PLK2 was found to be remarkably reduced in GBM in contrast with non-tumorous tissues, which may result from PLK2 methylation in GBM. However, low PLK2 expression seems to suggest a good prognosis for patients. Similarly, in the study by Xia et al., the researchers found that PLK2 methylation resulted in its reduced expression in GBM patients [16]. Furthermore, low PLK2 expression has been found to correlate with better prognosis and its possible use as a prognostic biomarker. Most importantly, the present study found that PLK2 may work as an independent predictor in the PLK family and demonstrates promise as a novel molecular target. In univariate and multivariate analysis, high contents of PLK2 were remarkably linked to OS time. Furthermore, high expression of PLK2 had a poor prognosis of patients with GBM. Therefore, PLK2 can be identified as an independent predictor in contrast with PLK1 in GBM. Current PLK2 inhibitors, ON1231320, has been demonstrated to be a selective repressor of PLK2, with no dampening activity against PLK1, PLK3 and PLK4. In contrast with the control group, PLK2 inhibitor treatment remarkably restrained tumor growth and there were no remarkable toxic effects in the treatment group [17]. ON1231210 is effective in different tumor cell lines, but not in non-tumorous human fibroblasts. The recent development of PLK2 ATP competitive inhibitors, has produces promising and specific molecules. Other examples of selective inhibitors of PLK2 have been also reported, thus demonstrating that efficient drug development strategy for the identification of PLK2 inhibitors have been reached by the scientific community [18]. This study is the first to use a PLK2 inhibitor in a glioma cell. Our results show that ON1231320 dampens GBM cell growth in a dose- along with time-dependent approach. Cleaved PARP is considered a marker of apoptosis [19]. The data illustrated that the expression of cleaved PARP was increased in U251MG and U87MG cells after ON1231320 treatment. Our data illustrated that ON1231320 dampened GBM cell growth in a dose- and time-dependent manner. *In vivo*, the inoculation with ON1231320 resulted in remarkable dampening of tumor growth in contrast with the control group. And then, ON1231320 shows its antitumor properties in glioma cells.

PLK3 is often lowly expressed in lung, head and neck cancers [20]. PLK3 is poorly studied in gliomas and GBM. PLK3 was found to be differentially expressed between IDH mutations carriers and wild-type carriers and was linked to LGG patient survival [21]. PLK3 regulates the cell cycle and is also a mediator of apoptosis [22]. This observation was consistent with the GSEA results. This study showed that PLK3 is remarkably upregulated in GBM, but the biological role of PLK3 in GBM needs further validation. PLK2 and PLK3 recognize a very similar consensus sequence [23]. However, the low number of PLK2 and PLK3 substrates identified so far could not be sufficient to highlight differences between two kinase. Moreover, α-synuclein C-terminal peptide is phosphorylated *in vitro* by PLK2 with a higher efficiency than by PLK3, thus suggesting some differences in substrates preference. Nevertheless, it must be considered that a similar consensus sequence is not sufficient to hypothesize redundant cellular functions for these kinases, as kinase specificity is the results of multiple mechanisms. Different spatial and temporal expression/activation of the two kinases or specific substrate binding sites could indeed account for distinct PLK2 and PLK3 cellular functions [18].

SAK-a and SAK-b are two isoforms of PLK4. PLK4 increases during the G1/S transition, then persists until late M phase, and finally decreases in early G1 phase. PLK4 is overexpressed in colon and breast cancers and downregulated in hepatocellular carcinoma [10].

Expression contents of PLK1 and PLK4 were elevated in contrast with non-tumorous mucosa [24]. This observation appears to be consistent with the PLK1/PLK4 co-occurrence described in Table 3. Wang et al. [25] observed elevated PLK4 expression in HGG patients, which was linked to poor prognosis. Zhang et al. discovered that the contents of PLK4 was remarkably correlated with glioma grade and inversely linked to overall survival of individuals with high-grade gliomas [26]. TMZ sensitivity was enhanced by depletion of PLK4 [26]. PLK4 inhibitor CFI400945 improves the sensitivity of GBM cells to TMZ [26].

PLK5 lacks a kinase domain in contrast with other PLK family members and therefore has no catalytic activity [10]. Interestingly, PLK5 is expressed primarily in the brains of humans. PLK5 is frequently silenced in astrocytoma and glioblastoma due to hypermethylation [27]. PLK5 in this study appeared to be potentially correlated with other PLK family members. Indeed, overexpression of PLK5 promotes tumor cell apoptosis. Thus, PLK5 is a kinase-deficient polo box domain-containing protein with exclusive neurological function and brain tumor suppressor activity [27].

Structurally, the general structure of PLK1-4 includes an N-terminal serine/threonine kinase domain along with a C-terminal polo-box domain (PBD), but the domain truncated in PLK5, without the T-loop [28]. The C-terminus contains two PBDs in Plk1-3 and Plk5, while there is only one PBD in PLK4 [29]. In Figure 5, there is a high positive correlation between PLK1 and PLK4. This may be due to the structural similarity between PLK1 and PLK4, which leads to their functional consistency. Pearson correlation analysis and mutual exclusivity analysis showed a remarkable positive correlation between PLK1 and PLK4. Zeng et al. also found the same conclusion in human non-small cell lung cancer [30]. The cross talk of PLK1 with PLK4 needs to be further demonstrated. PLK1 and PLK4 expression levels are higher in leukemia cells than in non-tumorous cells and have promise as being new targets for cancer therapy [28]. Therefore, theoretically combined dampening of PLK1 and PLK4 seems to be an effective treatment of GBM. It has been shown that the ATP-binding site of kinases is a target for the design of inhibitors that can be used to inhibit kinase. The kinase domain of PLK1 is similar in sequence to PLK2, PLK3 and PLK4, the development of a specific, ATP-competitive PLK1 inhibitor remains challenging. It is therefore conceivable that inhibitors of PLK1, scarcely discriminate between PLK1–PLK3. Unfortunately, PLK4 is not dampened by inhibitors of PLK1, and it is possible that PLK4 is very different in contrast with other PLK family members in terms of structure [7]. There is a need to further understand which gene is dominant in this correlation, to explore whether there is a common signaling cascade between PLK1 and PLK4, and to suppress the high expression of both PLK1 and PLK4 by inhibiting a target gene. Considering the expression of PLK4 in GBM, it may be possible to control GBM progression more effectively if PLK1-4 can be dampened simultaneously. The alteration of PLKs in GBM was mainly expressed in amplified form (Figure 3), which explains that PLKs are still mostly upregulated with increasing glioma grade. Furthermore, PLK alterations usually occur in primary GBM, suggesting an important role for PLKs in primary GBM.

P53 plays an indispensable role in transcriptional regulation of BP, such as DNA repair, cell cycle arrest, senescence, and apoptosis [31]. PLKs-associated KEGG cascade analysis also shows correlation with p53 signaling cascade. This means that the p53 signaling cascade could be the key signaling cascade in PLKs network. In addition, PLK2 may contribute to the poor prognosis of glioma patients through other cascades.

Herein, our results suggest that the high expression levels of PLK1/3/4 and low expression levels of PLK2/5 play important roles in the development of GBM. PLK family can be an important molecular marker to identify the malignancy of GBM. Furthermore, our findings suggest that PLKs are potential therapeutic targets for GBM. In particular, low PLK2 expression presents a potential prognostic marker for improving GBM patient survival and prognostic accuracy.

## MATERIALS AND METHODS

### ONCOMINE analysis

PLK transcription levels in various malignancies were analyzed using gene expression array datasets from ONCOMINE, an open cancer microarray data repository. The tumor analyses were in contrast with the non-tumorous tissue of the same kind. The PLK transcript contents in clinical cancer specimens were in contrast with those in non-tumorous controls using the student’s *t*-test, which yielded a *p*-value. 0.01 and 2 were chosen as the *p*-value cut-off and fold change, respectively.

### Gene expression profiling interactive analysis 2(GEPIA2) dataset

GEPIA2 is a newly established interactive web resource that employs a standard processing pipeline to explore the RNA sequencing expression data of 9,736 tumors along with 8,587 non-tumorous samples from the TCGA, as well as GTEx projects (http://GEPIA2.cancer-pku.cn/) [10]. For tumor/non-tumorous differential expression and correlation analysis, GEPIA2 was employed. The approach for estimating differential expression constitutes one-way ANOVA, with disease status (tumorous or non-tumorous) as the variable.

### cBioPortal

The cBio Cancer Genomics Portal (http://cbioportal.org) was created primarily to resolve the data integration challenges that large-scale cancer genomics research suffer [32]. We identified somatic mutations, transcript expression changes, along with copy number changes in a set of cases using OncoPrints. Based on the cancer types summary, various PLK alteration forms were observed in GMB, including mRNA low, mutation amplification, mRNA high, deep deletion, and multiple alterations. The difference between the altered and unaltered groups in the subtypes was determined via clinical comparison. To understand the mutual exclusivity between PLKs, the samples with a variation on a gene were used as a set to analyze whether two genes were mutually exclusive or co-occurring in a tumor by analyzing the sets corresponding to the two genes. A positive value here suggests that alterations in these genes co-occur in the same samples, while a negative value suggests that alterations in these genes are mutually exclusive and occur in different samples.

### TCGA data

We acquired 699 samples from TCGA, which included gender, WHO grade, age, and pathologic diagnosis. The molecular analysis results were obtained using the Genomic Data Commons (GDC) [33].

### CGGA data

The CGGA data resource (http://www.cgga.org.cn/) is a user-friendly web tool for data storage and analysis that allows you to explore brain tumor data sets consisting of over 2,000 samples from Chinese cohorts [34]. This study utilized CGGA tools for grade-related differential expression, and correlation analyses. In addition, 693 samples were downloaded for mRNA sequencing.

### HPA data

The Human Protein Atlas data resource (https://www.proteinatlas.org/) involves mapping all human proteins in cells, tissues, along with organs by incorporating multiple omics approaches, for instance antibody-centered imaging, mass spectrometry-centered proteomics, transcriptomics, as well as systems biology [35]. The HPA data set was adopted to verify PLKs at the translational level. The score of protein expression was calculated via immuno-histochemistry data that was manually rated for staining intensity (negative, weak, moderate, or strong) and the proportion of stained cells (25%, 25–75%, or >75%).

### Functional enrichment analyses

GO along with KEGG analyses were also adopted to assess the functional roles of the PLK network. The cluster Profiler program in R statistical software was adopted to conduct these gene functional enrichment analyses (The R Foundation, Vienna, Austria). *P* < 0.05 served as the cutoff for GO along with KEGG enrichment analyses. The findings of the GO along with KEGG assessments were visualized using the R tool GO plot.

### GSEA

GSEA (http://www.broadinstitute.org/gsea/index.jsp) constitutes a statistical approach for determining if an a priori defined list of genes exhibits remarkably different, concordant differences between two biological states. The GSEA v4.0 software was adopted to assess the normalized enrichment score (NES) along with false discovery rate (FDR) to validate the remarkable changes [36].

### Cox proportional hazards regression analysis

Univariate along with multivariate Cox proportional hazards regression assessments were adopted to explore predictive clinico-pathological variables. The hazard ratio for each category and its 95% CI are utilized to express the results for noteworthy prognostic indicators. *P* < 0.05 was regarded as statistically significant. R statistical program was used to conduct the statistical analysis.

### GeneMANIA in Cytoscape

GeneMANIA utilizes a vast database of functional relationship data to uncover other genes that are connected to a set of input genes. Protein along with genetic relationships, cascades, co-expression, co-localization, as well as protein domain similarity are all examples of association data [16]. GeneMANIA may be accessed using the Cytoscape app. Using GeneMANIA, we created a network of 100 genes connected to PLKs.

### Cell culture

The Chinese Academy of Sciences provided U251MG (Shanghai, China). The cells were propagated in DMEM (Gibco; Thermo Fisher Scientific, USA) enriched with 10% FBS (Gibco; Thermo Fisher Scientific, USA) under 37°C along with 5% CO_2_ conditions.

### Cell viability assay

The CCK8 kit was adopted to assess cell viability. To begin, 5000 cells/well were inoculated in 96-well plates. The cells were inoculated with diverse dosages of ON1231320 and propagated for 24, 48, and 72 hours after an overnight incubation. After the inoculation, each well was introduced with 100L DMEM and 10L CCK-8 and incubated for two hours. Finally, utilizing an Infinite M200 PRO plate reader, the absorbance was taken at 450 nm (Tecan, Switzerland).

### Colony forming cell assay

On 6-well culture plates, 500 cells/well were planted and grown in DMEM enriched with 10% FBS. The cells were inoculated with the appropriate agents and grown at 37°C along with 5% CO_2_ conditions for ten days. The colonies were then enumerated after being stained with 0.1 percent crystal violet. Three independent tests were performed on each group of colonies. ImageJ software was adopted to evaluate the data.

### Western blot assay

Cell lysing was done with the RIPA buffer (APPLYGEN, Beijing, China) enriched with a cocktail of protease inhibitor. Fractionation of proteins was done with a suitable dosage of gel (BioSci: 8012011), followed by blotting onto nitrocellulose membranes. After that, 3% BSA in TBST was adopted to block the membranes for one hour at RT (room temperature). Thereafter, we overnight inoculated the samples with the primary antibody of the matching antigen at 4°C, and then rinsed thrice in TBST. Afterwards, we inoculated the samples for one hour with the fluorescently linked secondary antibody at RT. Using the Odyssey infrared imaging equipment (LI-COR, Lincoln, NE), distinct protein bands were identified after three rinses in TBST. For sample loading and standardization, GAPDH was employed as an internal control.

### Immunohistochemistry

PLKs were stained immuno-histochemically on 5-m unstained tissue slices, which were subsequently deparaffinized, then re-hydrated. Tissue slices were treated for 30 minutes at 100°C in a steamer harboring 10 mM citrate buffer (pH 6.0) to assess antigenicity. To suppress activity of endogenous peroxidase, the segments were sub-merged in ethanol enriched with 3% hydrogen peroxidase for 20 minutes. Sections were inoculated overnight at 4°C with a 1:100 dilution of primary anti-PLKs (Santa Cruz, CA, USA). The tissue slices were then rinsed in PBS and inoculated for 30 minutes with an anti-rabbit secondary antibody before being inoculated with the streptavidin horseradish peroxidase complex. DAB was used to develop the sections, which were then counterstained with hematoxylin. At 400 magnifications, 15–20 fields of the sections were examined. Four grades categorized the fraction of positively-stained tumor cells: >30% positive tumor cells (3); 10–30% positive tumor cells (2); <10% positive tumor cells (1); no positive tumor cells (0). Four grades categorized the intensity of staining: no staining (0), light-yellow-weak staining (1); yellowish brown-moderate staining (2), and brown-strong staining (3). The staining index was computed as the fraction of positively stained tumor cells multiplied by the intensity of staining. High PLKs expression was described as a staining index score >4, whilst low expression was characterized as a staining index score 2, and mid expression was defined as others score.

### Nude mouse xenograft

5 × 10^6^ U87 cells were subcutaneously inoculated into the right axillae of 6 weeks old BALB/c Nude female mice. Once the tumors have grown in the mice for 2 weeks, we stratified the mice at random into treatment groups (*n* = 4) and were given daily, either placebo, or the specified dosages of ON1231320. The formula (long × short2)/2 was employed to compute tumor volumes.

### Statistical analysis

For all statistical analyses, SPSS v20.0 was utilized. The unpaired Student's *t*-test was adopted to uncover differentially expressed genes. For the statistical examination of the correlation between two independent variables, the 2 test was used. The survival distributions were estimated using Kaplan-Meier survival analysis. Using GraphPad Prism, the log-rank approach was adopted to assess the statistical significance of the stratified survival groups.

### Data accessibility

These data were derived from the following resources available in the public domain: TCGA database (https://portal.gdc.cancer.gov/) and CGGA database (http://www.cgga.org.cn/).
